# Video Game Rehabilitation for Outpatient Stroke (VIGoROUS): protocol for a multi-center comparative effectiveness trial of in-home gamified constraint-induced movement therapy for rehabilitation of chronic upper extremity hemiparesis

**DOI:** 10.1186/s12883-017-0888-0

**Published:** 2017-06-08

**Authors:** Lynne V. Gauthier, Chelsea Kane, Alexandra Borstad, Nancy Strahl, Gitendra Uswatte, Edward Taub, David Morris, Alli Hall, Melissa Arakelian, Victor Mark

**Affiliations:** 1 The Ohio State University, Department of Physical Medicine and Rehabilitation, Division of Rehabilitation Psychology, 480 Medical Center Drive, Columbus, OH 43210 USA; 2 Department of Physical Therapy, College of St. Scholastica, 1200 Kenwood Ave, Duluth, MN 55811 USA; 3 Providence Medford Medical Center, 1111 Crater Lake Ave, Medford, Oregon, 97504 USA; 4 Department of Psychology; UAB Department of Psychology, University of Alabama at Birmingham, Campbell Hall 415, 1530 3rd Avenue South, Birmingham, AL 35294-1170 USA; 5 Department of Physical Therapy; UAB Department of Physical Therapy, University of Alabama at Birmingham, 1720 2nd Avenue South, School of Health Professions Building 360X, Birmingham, AL 35294-1212 USA; 6 Department of Physical Medicine and Rehabilitation, University of Alabama at Birmingham, 1720 2nd Avenue South, Birmingham, AL 35294-7330 USA; 7 Department of Neurology, University of Alabama at Birmingham, 1720 2nd Avenue South, Birmingham, AL 35294 USA

**Keywords:** Protocol, Research design, Randomized controlled trial, CI therapy, Constraint-induced movement therapy, Rehabilitation, Video game, Stroke, Virtual reality, Motor, Hemiparesis

## Abstract

**Background:**

Constraint-Induced Movement therapy (CI therapy) is shown to reduce disability, increase use of the more affected arm/hand, and promote brain plasticity for individuals with upper extremity hemiparesis post-stroke. Randomized controlled trials consistently demonstrate that CI therapy is superior to other rehabilitation paradigms, yet it is available to only a small minority of the estimated 1.2 million chronic stroke survivors with upper extremity disability. The current study aims to establish the comparative effectiveness of a novel, patient-centered approach to rehabilitation utilizing newly developed, inexpensive, and commercially available gaming technology to disseminate CI therapy to underserved individuals. Video game delivery of CI therapy will be compared against traditional clinic-based CI therapy and standard upper extremity rehabilitation. Additionally, individual factors that differentially influence response to one treatment versus another will be examined.

**Methods:**

This protocol outlines a multi-site, randomized controlled trial with parallel group design. Two hundred twenty four adults with chronic hemiparesis post-stroke will be recruited at four sites. Participants are randomized to one of four study groups: (1) traditional clinic-based CI therapy, (2) therapist-as-consultant video game CI therapy, (3) therapist-as-consultant video game CI therapy with additional therapist contact via telerehabilitation/video consultation, and (4) standard upper extremity rehabilitation. After 6-month follow-up, individuals assigned to the standard upper extremity rehabilitation condition crossover to stand-alone video game CI therapy preceded by a therapist consultation. All interventions are delivered over a period of three weeks. Primary outcome measures include motor improvement as measured by the Wolf Motor Function Test (WMFT), quality of arm use for daily activities as measured by Motor Activity Log (MAL), and quality of life as measured by the Quality of Life in Neurological Disorders (NeuroQOL).

**Discussion:**

This multi-site RCT is designed to determine comparative effectiveness of in-home technology-based delivery of CI therapy versus standard upper extremity rehabilitation and in-clinic CI therapy. The study design also enables evaluation of the effect of therapist contact time on treatment outcomes within a therapist-as-consultant model of gaming and technology-based rehabilitation.

**Trial registration:**

Clinicaltrials.gov, NCT02631850.

## Background

Clinical practice guidelines recommend outpatient rehabilitation for stroke survivors who remain disabled after discharge from inpatient rehabilitation [1]. Although these guidelines recommend that the majority of stroke survivors receive at least some outpatient rehabilitation [2], many cannot access long-term care [3]. Among those individuals who do undergo outpatient rehabilitation, the standard of care for upper extremity rehabilitation is suboptimal.

In an observational study of 312 rehabilitation sessions (83 occupational and physical therapists at 7 rehabilitation sites), Lang and colleagues [4] found that functional rehabilitation (i.e., movement that accomplishes a functional task, such as eating, as opposed to strength training or passive movement) was provided in only 51% of the sessions of upper extremity rehabilitation, with only 45 repetitions per session on average. This is concerning given that empirically-validated interventions incorporate higher doses of active motor practice [5–7]. Additionally, functional upper extremity movements are most likely to generalize to everyday tasks [8], an aspect of recovery that is critically important to patients and their families [9–11]. Yet, passive movement and non-goal-directed exercise are more frequently administered [4].

There appear to be at least two critical elements required for successful upper extremity motor rehabilitation: 1) motor practice that is sufficiently intense and 2) techniques to carryover motor improvements to functional activities. Carry-over techniques to increase a person’s use of the more affected upper extremity for daily activities are extremely important for rehabilitation and appear necessary for structural brain change [12–15]. When rehabilitation incorporates these techniques, there is substantially improved improvement in self-perceived quality of arm use for daily activities [12, 16]. Carry-over techniques enable the patient to overcome the conditioned suppression of movement (learned nonuse) characteristic of chronic hemiparesis [17]. Techniques include structured self-monitoring, a treatment contract, daily home practice of specific functional motor skills, and guided problem-solving to overcome perceived barriers to using the extremity [18].

Constraint-Induced Movement therapy (CI therapy) has strong empirical backing [5, 19] and combines high-repetition functional practice of the more affected arm with behavioral techniques to enhance carry-over [13, 18]. CI therapy produces consistently superior motor performance and retention of gains versus standard upper extremity rehabilitation [20, 21], particularly when it includes the critically important carry-over (transfer package) techniques [12]. When compared to other equally intensive interventions (i.e., equal hours of training on functional tasks), CI therapy with carry-over (transfer package) techniques has also shown enhanced carry-over of clinical gains to daily activities [12, 13, 22–24] that are retained for at least 2 years [19, 25–28].

Despite its inclusion in best practice recommendations [29, 30], CI therapy is available to only a very small minority of those who could benefit from it in the US. CI therapy is not typically covered by insurance and the 30+ hours of assessment and physical training cost upwards of $6000. Access barriers for the patient include limited transportation and insurance coverage, whereas therapists may have difficulty accommodating the CI therapy schedule [31, 32]. Access barriers aside, CI therapy has also been plagued by a variety of misconceptions regarding use of restraint and the transfer package. Most iterations of CI therapy employ use of a restraint mitt to promote use of the affected arm, which is viewed by many patients and clinicians as excessively prohibitive [32]. Yet, literature demonstrates that restraint is not specifically required to achieve positive outcomes [33, 34]. Moreover, the transfer package, a component found to be critical [13, 14], is omitted from the majority of research studies on CI therapy [35].

To address transportation barriers, a telerehabilitation model of CI therapy delivery (AutoCITE) has been tested. AutoCITE is a large specialized motor apparatus (not commercially available, cost not established) that was installed in patients’ homes to enable therapeutic manipulation of actual objects with continuous video monitoring via Internet. This telerehabilitation approach demonstrated efficacy approximately equivalent to that of in-clinic CI therapy [36–38], thus establishing the feasibility of utilizing technology to deliver CI therapy remotely. However, this system involved specialized equipment at a high cost and did not become available outside a research setting.

To more fully address the barriers to accessing CI therapy and to counter the misconceptions surrounding CI therapy, a patient-centered treatment approach was developed that incorporated the high-repetition practice and carry-over strategies from CI therapy, while reforming non-patient-centric elements of the protocol that lack strong empirical support (i.e., the restraint). To deliver engaging high-repetition practice, a Kinect-based video game was created that can accommodate a wide range of motor disability, can be customized to each user, and automatically progresses in difficulty as the individual’s performance improves (termed “shaping” in the CI therapy literature). A player’s body movements drive game play (there is no external controller), which makes the game easy to use for those who may be unfamiliar with technology. To date, such high-repetition practice through motor gaming [39] has shown initial promise compared to traditional clinic-based approaches [40]. To promote increased use of the weaker arm, a smart watch biofeedback application is utilized in lieu of the restraint mitt. This application counts movements made with the weaker arm and provides alerts when a period of inactivity is detected. Previous approaches for providing CI therapy in the home and reducing the amount of therapist effort have been carried out [36–38, 41]. These approaches automated the delivery of training and permitted remote supervision of the training via an Internet-based audio-visual link, but did not embed the training within the context of a video game, rely on manipulation of virtual objects, or incorporate a patient-centric substitute for the mitt.

Initial evidence from a pilot trial of this system (Borstad A, Crawfis R, Phillips K, Pax Lowes L, Worthen-Chaudhari L, Maung D, et al.: In-home delivery of constraint induced movement therapy via virtual reality gaming is safe and feasible: a pilot study, submitted) suggests that improvements in motor speed, as measured by Wolf Motor Function Test (WMFT) performance time [42], an outcome of prime importance to stroke survivors, are approximately equivalent to those reported in the traditional CI therapy literature [5, 13, 19, 25]. Qualitative data reveal that the technology is accepted irrespective of age, technological expertise, ethnicity, or cultural background. Thus, this technology has the potential to address the main barriers to adoption of CI therapy, while reducing the cost of care. A randomized clinical trial is now required to provide Level 1 evidence of the comparative effectiveness of this novel model of CI therapy delivery. Data from this trial will enable individuals with motor disability to evaluate whether a home-based video game therapy has the potential to help them meet their rehabilitation goals compared to in-clinic CI therapy and traditional approaches. By combining novel gaming elements with the transfer package from CI therapy, this trial will also address a major limitation of rehabilitation gaming interventions that have been tried to date: extremely limited emphasis on carry-over of training to daily activities.

The primary objective of this trial is to compare the effectiveness of two video game-based models of CI therapy versus traditional clinic-based CI therapy versus standard upper extremity rehabilitation for improving upper extremity motor function. One video gaming group will match the number of total hours spent on the CI therapy transfer package, but will involve fewer days of therapist-client interaction (4 versus 10); the other will match the number of interactions with a therapist to that of clinic-based CI therapy using video consultation between in-person sessions and, as such, will involve more therapist contact hours spent focusing on the transfer package. The secondary objective of this project is to promote personalized medicine by examining individual factors that may differentially influence response to one treatment versus another.

## Methods/design

### Study setting

Study screenings, interventions, and assessments are conducted at four separate sites in the United States, each an outpatient medical facility. The four study sites include Ohio State University (Columbus, OH), Ohio Health (Columbus, OH), Providence Medford Medical Center (Medford, OR), University of Alabama (Birmingham, AL). Interventions are completed by licensed physical/occupational therapists or individuals with appropriate clinical training (e.g., occupational therapy assistants, graduate students) under the supervision of a licensed physical or occupational therapist. Game-play and home-practice occurs independent of the therapist’s presence and is performed in the home environment.

### Eligibility criteria

#### Inclusion

Broad enrollment criteria will be employed to include a representative sample of patients with upper extremity motor impairment. In order to be eligible to participate in this study, an individual must meet all of the following criteria:

- Ability to demonstrate informed consent to participate in research (see “Informed Consent” below).
- Expressed willingness to attempt to comply with all study procedures and attend all study-related visits.
- Age ≥ 18.
- Demonstrated ability to comprehend English and participate in basic elements of the study, as evidenced by ability to follow one-step commands.
- Community-dwelling.
- Experienced a stroke (of any etiology) resulting in mild to moderate hemiparesis at least six months prior (defined by criteria in Table 1).
- Can independently operate the gaming system (those with severe cognitive impairments can usually achieve this).
- Corrected vision of at least 20/70 as assessed by their ability to identify game objects on the monitor from 5 ft away.

**Table 1:** Minimum Active and Passive Range of Motion (ROM) required for participation

	Shoulder	Elbow	Wrist	Fingers	Thumb
Minimum passive ROM	Flexion ≥90° Abduction ≥90° External rotation ≥45°	Extension to ≥150°	Extension ≥0° Forearm supination/pronation ≥45°	MCP extension to ≥145°	Extension or abduction of thumb ≥10°
Minimum active ROM	Flexion ≥45° and Abduction ≥45°	Extension ≥20° from a 90° flexed starting position	Extension ≥10° from fully flexed starting position	Extension MCP and (PIP or DIP) joints of at least 2 fingers ≥10°	Extension or abduction of thumb ≥10°

*MCP* metacarpophalangeal, *PIP* proximal interphalangeal, *DIP* distal interphalangeal

Individuals will be enrolled irrespective of ethnicity, gender, native language (assuming basic comprehension of English), or mobility; thus, this work will include individuals who have historically been excluded from CI therapy trials (e.g., wheelchair users, those with substantial cognitive impairment, those with balance impairments) to increase the generalizability of the results.

Data regarding current medication prescription and usage is collected during the in-person screening phase. Participants are asked to limit changes to spasticity-modifying medications during participation in the study. Participants are asked about the use of anti-spasticity agents during in-person screening. Participants who report receiving botulinum toxicity injections may not participate in pre-testing or intervention within 12 weeks from the last injection, but may begin participation 12 weeks after their last botulinum toxin injection as long as they refrain from additional Botox therapy during the study intervention phase and 12 weeks prior to follow-up testing. If the participant is using other antispasticity agents (i.e. oral Baclofen), this information is noted in the participant’s file but does not exclude them from participating in the study.

#### Exclusion

Although CI therapy has been successfully offered (with adaptations) for those with more severe motor impairments [43], this trial excludes these individuals because substantial modifications to the protocol would be required.

An individual who meets any of the following criteria is excluded from participation in this study:

- Concurrent participation in other experimental upper extremity rehabilitation trials.
- Concurrent participation in other outpatient rehabilitation for their upper extremity during the treatment phase(s) of the study.
- Botox within 3 months prior to beginning study-related treatments (confound).
- Substantial use of the more-affected arm in daily life (Motor Activity Log [MAL] score at screening >2.5).
- Major medical issues such as upcoming surgery or procedures that would interfere with study treatments or make intensive rehabilitation difficult to tolerate. Potential participants will be given the option to self-exclude if they have pain that would be significantly aggravated by participation in the protocol and would limit ability to complete treatment per protocol.

### Interventions

An advisory board that comprises therapists, caregivers, and stroke survivors assisted to formulate the study design. In particular, these stakeholders provided insight into specific concerns and needs relevant to the stroke community. The relevant questions posed by the stroke community were: Is home-based CI therapy through gaming as effective as in-clinic CI therapy? Can therapist time be used more efficiently by supplementing in-clinic practice with in-home gaming CI therapy? Will more frequent contact with a therapist (whom they feel holds them accountable for completing the therapy protocol) lead to better outcomes? The below comparison groups will address these questions using appropriate controls. Stakeholders nearly unanimously reported preferring the more intense and compressed treatment delivery schedule of CI therapy compared to a more distributed treatment delivery schedule, but also requested a slight reduction in the treatment days per week from traditional CI therapy (10 days in 2 weeks) to better accommodate caregiver schedules. Thus, all interventions occur over a 3 week period. A target of 224 participants will be randomized to one of four intervention groups (56 per group). See Table 5 for an overall depiction of the study timeline, discussed in more detail in the Participant Timeline section***.***

#### Group 1: Traditional in-clinic CI therapy

This group follows the established method of delivering CI therapy, as documented by Morris, Taub, and colleagues [18]. Participants randomized to this group receive ten 3.5-h sessions of in-clinic traditional CI therapy with a therapist. They agree to wear a restraint mitt for a target of 10 h daily. Participants receive 30 h of massed practice with shaping (50% of this time spent in active movement) and 5 h of transfer package techniques emphasizing carry-over of motor gains to daily activities (more detail below).

*Massed motor practice with shaping* occurs for 3 h each treatment day on 10 treatment days within a 3-week period. Five to nine tasks per day are administered, each consisting of up to ten 30–120 s trials. Tasks are selected such that they are well within a participant’s capability, but are not optimally performed by a participant. Treatment time consists of 50% active motor practice and 50% rest/task set-up, for a target of 15 h active motor practice over the course of the intervention. Trial-level performance feedback (e.g., number of repetitions in a given task time, time to complete a fixed number of repetitions) is provided to the participant following every trial. Verbal encouragement and/or coaching is provided on at least 80% of trials. Task difficulty is shaped (progressed) systematically (e.g., when the average of the most recent 5 trials exceeds the average of the previous 5 trials). See Shaping Procedure document published on the PI’s ResearchGate site (link below in *Availability of Data and Materials* section) for a more detailed account of the shaping procedure.

*The transfer package of carry-over techniques* consists of the following 5 elements (for more detail, see Morris, D., Taub, E., & Mark, V. (2006) [18]).

1. *Treatment Contract:* Completing the Treatment Contract takes approximately 45 min and involves learning the elements of the intervention, establishing a formal agreement to adhere to the different elements of CI therapy, goal setting, and outlining in detail which activities of daily living will be performed exclusively with the more affected upper extremity versus with both upper extremities versus with the less affected upper extremity exclusively. The participant maintains a Home Achievement Record for daily self-monitoring of arm use for pre-specified daily activities that are agreed to during completion of the Treatment Contract. Adherence to the contract is reviewed on each of the subsequent 9 treatment days. Guided problem-solving is carried out as necessary to enhance use of the weaker arm for daily activities. A Caregiver Contract provides guidance on when it is appropriate for the caregiver to assist with ADLs and on how to provide effective encouragement.
2. *MAL:* The MAL assesses the amount and quality of upper extremity arm use for 28 activities of daily living. The full MAL is administered on Days 1 and 6, whereas half of the MAL is administered on other treatment days (total of 6 administrations). The therapist uses guided problem-solving with the participant regarding strategies to improve functional use of the more affected upper extremity on at least 2 items from the MAL, per administration. The “say-do-check” problem-solving procedure employed here is based on the work of Skidmore and colleagues (2011) [44]. The “say-do-check” procedure emphasizes eliciting strategies from the participant, objectively testing the effectiveness of these strategies, and revising the plan as needed. See Guided Problem Solving document on ResearchGate for more detail.
3. *Home Skill Assignment:* Participants practice 10 functional motor tasks that are collaboratively agreed upon (e.g., play drums, practice golf swing, sort silverware) for 30 min per treatment day, beginning after the second treatment day. Activity is recorded by the participant and is discussed with the therapist daily.
4. *The restraint mitt* is worn on the less affected upper extremity for a target of 10 h per day (both in and outside of treatment) on all treatment days to encourage using the more affected arm. A motion sensor with LCD display is placed within the mitt to provide the participant with immediate feedback on how long the mitt was worn and to enable therapists to monitor treatment adherence.
5. *MAL in follow-up:* To promote enhanced carry-over and retention, 4 weekly teleconsultation sessions are scheduled after treatment. These sessions involve telephone administration of the MAL with problem-solving.

#### Group 2: In-home gaming CI therapy + in-clinic therapist consultation

This group utilizes in-home gaming CI therapy in conjunction with in-clinic consultation with a therapist throughout the intervention period. The following components of the intervention will be included: 1) high repetition motor practice through use of game-based CI therapy, 2) a transfer package of carry-over techniques, and 3) using smart watch technology to promote using the more affected upper extremity.

*High repetition motor practice* is achieved through in-home game play. Participants agree to play the game for 15 h over three weeks (ten treatment days; approximately 1.5 h of game play daily). Participants are encouraged to break play into at least 3 separate play sessions per day to allow appropriate rest. Participants are also encouraged to complete any missed game play during “off” days. Instruction to play the game is provided for approximately 30 min on the first treatment day. The gaming system logs play times and they are stored on a cloud-based server to enable the therapist to monitor treatment compliance. To enable including participants in rural or urban areas regardless of Internet access, participants assigned to the gaming groups are lended mobile hotspots during the treatment period. On subsequent days, the therapist checks participants’ adherence to game play and uses guided problem-solving to address compliance when necessary.

**Fig. 1:**
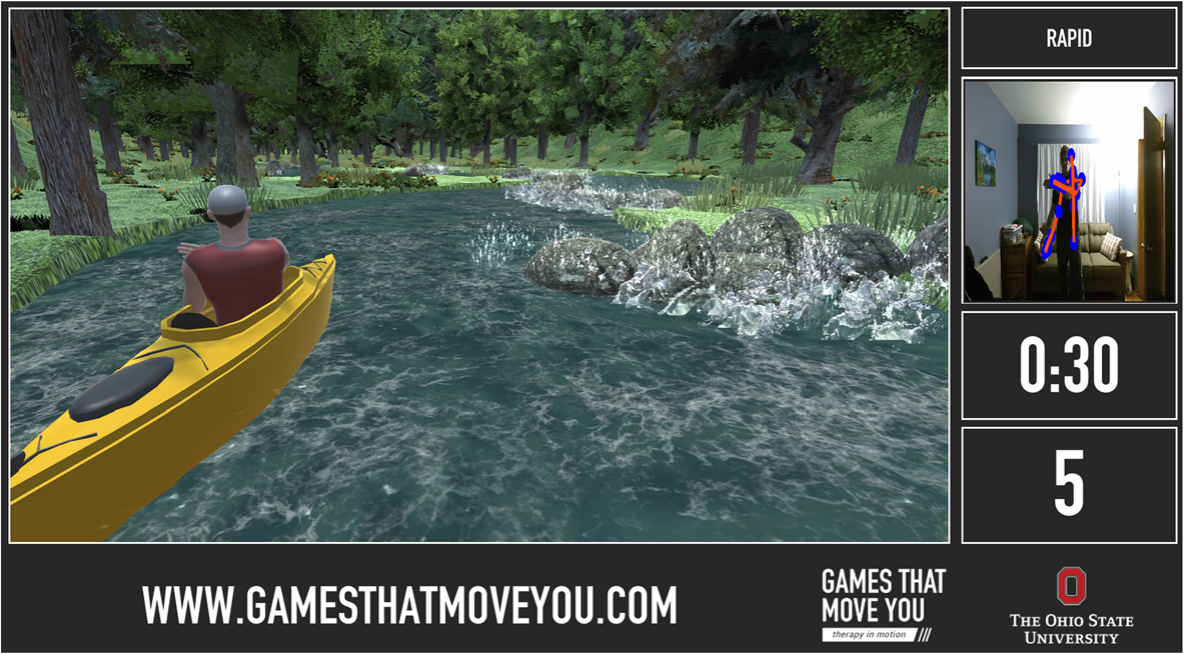
Screen capture of the Recovery Rapids gaming environment

Game play is driven solely by movements made with the more affected upper extremity (Table 2). The CI therapy principle of “shaping” is incorporated into the game, such that movements are automatically made more difficult as a person’s range of motion improves. For example, to move the game character forward, the user performs a row gesture consisting of shoulder flexion/extension with elbow extension. The software will initially require just 30 degrees of shoulder flexion to trigger forward movement; however, if a person is able to consistently demonstrate 60 degrees of flexion with almost full extension, the software will require that the user perform the movement to his/her capacity. The gaming environment is shown in Fig. 1.

**Table 2:** Upper extremity motions corresponding to game actions

Motion	Game action elicited
Shoulder flexion/extension with elbow extension	Row kayak down river
Shoulder abduction with elbow extension	Steer kayak toward hemiparetic side
Horizontal shoulder adduction across midline	Steer kayak away from hemiparetic side
Elbow flexion/extension	Catch fish with a net
Elbow flexion/extension and grasp/release	Collect bottles from a river
Forearm supination with shoulder flexion and elbow extension	Catch parachute to receive supplies
Finger flexion/extension and thumb abduction/adduction, shoulder flexion to position hand over target	Pick fruit from bushes
Forearm supination with shoulder flexion to position hand over target	Turn over card
Wrist extension with shoulder flexion to position hand over target	Flick letters in word puzzle

*The transfer package of carry-over techniques* for Group 2 includes the same elements of the transfer package of traditional CI therapy (Treatment Contract, MAL, Home Skill Assignment), with the exception of the mitt restraint being replaced with a smart watch biofeedback app (see below). Administrating the Treatment Contract and Caregiver Contract is identical to Group 1 and occurs at the beginning of the first treatment day. The Home Achievement Record of the Treatment Contract is reviewed on each subsequent treatment visit and guided problem-solving is carried out as necessary to enhance use of the weaker arm for daily activities. Similarly, the Home Skill Assignment is identical to Group 1, but begins on the first treatment day.

The MAL is administered on each treatment day, but is done so via a computer application. One half of the MAL is completed at home by the participant on each treatment day. When a participant reports having not engaged in an activity, a problem-solving module is presented whereby the participant selects a potential strategy to enhance success from a pre-derived list of 2–3 strategies. Participant responses are stored on a cloud-based server so they can be remotely accessed by a therapist. The therapist reviews the MAL responses and uses guided problem-solving on at least 2 items on each subsequent treatment day. The *MAL in follow-up* is administered weekly for 4 weeks via REDCap (Research Electronic Data Capture) survey, an Internet-based data collection system (see Data Collection & Management). Participants without internet access (Hotspots are returned at post-test) complete paper and pencil versions of the MAL and mail them in.

*Smart watch biofeedback technology* is utilized to promote use of the more affected upper extremity during daily activities in lieu of the mitt. The decision to replace the mitt with a PebbleTime_TM_ smart watch app was informed by questionable importance of the mitt component of CI therapy [33, 34], poor compliance with mitt use in a previous pilot study (Borstad A, Crawfis R, Phillips K, Pax Lowes L, Worthen-Chaudhari L, Maung D, et al.: In-home delivery of constraint induced movement therapy via virtual reality gaming is safe and feasible: a pilot study, submitted), and expression of a strong preference for the app by the participants. The smart watch app uses a tri-axial accelerometer to record the number of movements made with the weaker arm. It provides approximate movement counts and alerts the user when a ten-minute period of inactivity is detected. The smart watch is worn on the less affected arm for 2 days prior to treatment initiation to establish a target for use of the weaker arm. Participants are then instructed to wear the watch on the weaker arm during waking hours throughout the intervention and to record their daily movement counts each evening before bed for later review by the therapist. By doing so, participants aim to utilize their more affected arm to the same extent during the trial as they used their less affected arm prior to the trial.

Game-play instruction and the above carry-over techniques are carried out over 4 therapist consultation sessions. In-person consultations occur approximately on days 1, 3, 5, 8. The initial consultation is 2 h and involves education on critical components of the intervention, customizing game play, teaching use of the technology, establishing the treatment contract, and performing guided problem-solving to address barriers to arm use. Subsequent sessions are one-hour and involve review of transfer package elements and guided problem-solving to promote increased affected arm use for daily activities.

#### Group 3: In-home gaming CI therapy + online therapist consultation

Treatment intervention for Group 3 is identical to Group 2, with the addition of supplemental videoconferencing with therapists. Group 3 participants receive video consultation sessions on the 6 treatment days that in-person therapist consultation does not occur (to equate the number of therapist consultation sessions between Group 1 and Group 3). The first video consultation session (day 2) is 1 h in length and each subsequent video consultation session (days 4, 6, 7, 9, 10) is 20 min for a total of 4 additional hours of teleconsultation. The Health Insurance Portability and Accountability Act (HIPAA) compliant videoconference program Bluejeans is utilized to facilitate secure communication for Group 3 teleconsultation. Ten total therapist encounters occur, equating total therapist encounters with that of traditional in-clinic CI therapy (Group 1). Video consultation sessions focus on review of transfer package elements and guided problem-solving to promote increased affected arm use for daily activities. Protocol adherence is reviewed and additional problem-solving is employed to improve compliance when needed. As with Group 1, the *MAL in follow-up* with guided problem-solving is administered via phone for 4 weeks post-intervention.

#### Group 4: In-clinic standard upper extremity rehabilitation

Group 4 completes five hours of in-clinic standard upper extremity rehabilitation over four sessions (same in-clinic schedule as Group 2). The intervention incorporates activities that are typically used in clinical rehabilitation settings. The standard care protocol was established to standardize “traditional care” based on responses from several practicing physical and occupational therapists regarding activities that they would incorporate into the care of individuals with mild/moderate upper extremity hemiparesis. The approach combines neuromuscular reeducation, functional training, progressive strengthening, and teaching/review of home programming. This protocol consists of 25 min of evaluation, 50 min of neuromuscular reeducation, 100 min of functional training, 70 min of progressive strengthening, and 55 min of teaching/reviewing home programming over the course of 4 sessions.

The relative time allocated to each activity reflects an average of the responses provided by the therapists. Following a 25 min evaluation on Day 1, the following components of the intervention are delivered: 1) neuromuscular re-education (20 min on Day 1, 10 min daily thereafter), 2) functional training (25 min all treatment days), 3) progressive strengthening (25 min on Day 1, 15 min thereafter), and 4) review/adjustment/teaching of home program (25 min on Day 1, 10 min thereafter). The activities incorporated into the treatment are designed to meet the specific needs of the participant (e.g., work to improve their body structure challenges, activity limitations, and participation goals). The protocol is standardized regarding activity categories, activity selection, activity intensity target level, and progression principles. It is expected that several trials of a given activity category are accomplished during specified time frames; it is expected that the number of trials may vary between participants based on their movement and endurance capabilities.

For the active procedures of neuromuscular re-education, functional training, and progressive strengthening, the target for exercise intensity is 4 (somewhat hard) on the Borg CR10 Rating of Perceived Exertion Scale (Table 3 below). Therapists work with the patient to ensure that activities are conducted with high quality and grade the difficulty of the activity so that the participant is consistently working at a level 4 on the Borg CR10 Rating of Exertion Scale. In subsequent visits, therapists adjust the activity grade (up or down) to achieve exertion of level 4 of the Borg CR10 Scale.

**Table 3:** Borg CR10 Rating of Perceived Exertion Scale

Classification	Descriptor
0	Nothing at all
0.5	Very, very light
1	Very light
2	Fairly light
3	Moderate
4	Somewhat hard
5	Hard
6	-
7	Very Hard
8	-
9	-
10	Very, very hard (maximum)

A home program is designed for all participants on the first visit. This home program consists of Thera-Band strengthening exercises to be done over a 15 min time period twice daily (total of 30 min daily) for the first 10 days that they are not receiving in-clinic therapy. Therapists design this program based on the participant’s skill with the exercises and the appropriate level of exertion (4 on the Borg CR10 Scale). During subsequent clinic visits (i.e., visits 2–4), therapists provide written instructions for exercises and review and modify the exercise program, as appropriate. Therapists may, at their discretion, give general recommendations for Group 4 clients to use the more affected arm outside of therapy (e.g., “try to use your weaker side more for dressing”). They will not engage the client in guided problem-solving or CI therapy transfer package activities (e.g., examining a client’s daily routine in detail), as these interventions are not typically utilized during standard care. The way in which therapists promote carry-over in Group 4 is thus much less specific/focused, less patient-centered, and less task-oriented than in Groups 1–3.

After 6 month follow-up testing, participants in Group 4 cross-over to a gaming condition that is identical to Group 2, except they will only receive a single 2-h consultation with a therapist instructing them on use of the system and educating them on transfer package components. This crossover design is ethically responsible and will enable the team to test the feasibility and initial efficacy of a stand-alone implementation of in-home gaming CI therapy, as well as to determine the effect of varying amounts of therapist contact on game-based rehabilitation outcomes [2 h (Group 4 crossed over) versus 5 h (Group 2) versus 9 h (Group 3)].

### Relevant group comparisons

The clinic-based CI therapy arm is the standard against which video game-based CI therapy will be compared (Group 1 versus Groups 2 and 3). Comparison between Groups 1 and 2 controls for time spent in active motor practice between in-clinic versus gaming CI therapy modalities. Comparison between Groups 1 and 3 controls for the number of encounters with a physical therapist between in-clinic versus gaming CI therapy. Comparison between Group 4 cross-over, Group 2, and Group 3 will determine the extent to which therapist contact influences treatment response of a home-based intervention. Comparison between Groups 2 and 4 will demonstrate whether game-based treatment between therapy sessions enhances motor outcomes, controlling for total face-to-face time with a physical therapist. All treatment materials relevant to each group can be accessed open source on the PI’s ResearchGate account.

### Hypotheses

We hypothesize that Groups 1–3 will not differ in response to treatment and that this response will be superior to that of Group 4 on both the MAL and the WMFT. We also anticipate that Groups 1–3 will show better retention of treatment gains than Group 4.

### Standardization of intervention

To ensure data quality and consistent administration of interventions across study arms and among all therapists, extensive training is conducted with interventionists. All sites received in-person training in the study procedures by Lynne V. Gauthier, Ph.D. (PI) and Alexandra Borstad, Ph.D., PT, NCS (Co-I). Additional training is provided as needed. Standardized checklists and treatment forms are utilized to ensure consistent documentation and to guide the therapist in adhering to the protocol.

To ensure fidelity of each intervention and reduce protocol drift, treatments are video recorded. Due to the extensive treatment time for Group 1, only select portions of this treatment will be recorded (days 1, 2, 6, and 10). These recorded sections include 2 shaping tasks and the entirety of the transfer package. Video recording instructions and listing of specified recording activities can be viewed on ResearchGate. A randomly selected subsample of recordings is reviewed by an independent rater for adherence to protocol. Therapists are periodically informed of their adherence and the PI is notified of any instances in which retraining is required. Relative time spent on each element of the intervention is logged in the project database, along with the name of the treating therapist. Although some variation is to be expected based on the individual needs of the client (e.g., one client may require longer to master game play than another), if systematic variations or <90% adherence to study protocol is observed for a particular therapist, that therapist receives personalized retraining. Persistent noncompliance by staff will be addressed by retraining and additional monitoring. If retraining is unsuccessful, noncompliance may result in termination. Noncompliance by staff that places participants at risk (e.g., violation of confidentiality) may be addressed by termination of staff. Noncompliance by investigators or staff will be reported to the Institutional Review Board (IRB).

Participant compliance with in-home portions of the study interventions is objectively recorded whenever possible. For example, an accelerometer device within the restraint mitt (Group 1) tracks wear time. Participants and their caregivers record compliance with use of the restraint mitt to cross-reference with the objective recordings or in case of technical failure. Game play data and computerized MAL administrations are stored both locally and on a cloud-based server with timestamp information that enables precise calculation of active play time. Use of the smart watch biofeedback device and performance of home-practice tasks is logged by the participant on study forms. Participant adherence to each element of the protocol is documented.

### Outcomes

There are three evaluation points in this study protocol: pre-treatment, post-treatment, and 6-months post-treatment. Individuals randomized to Group 4 complete additional post-treatment testing after they complete the crossover treatment condition (see Table 5). Table 4 lists the measures administered throughout the course of the study. The primary endpoint is immediately post-treatment (effectiveness of intervention). Six-month follow-up is considered as a secondary endpoint, as retention is likely more strongly influenced by factors outside of the experimental protocol (e.g., interim care).

**Table 4:** Testing measures collected at each time point

Datum	Primary	Secondary	Addresses	Pre	Post	6mo
Motion Capture via wearable sensors		X	Nonuse for daily activities	X	X	X
Motor Activity Log (MAL)	X		Nonuse for daily activities	X	X	X
WMFT Performance Time	X^a^		Motor Speed	X	X	X^b^
Grip strength of WMFT	X^a^		Weakness	X	X	X^b^
Kinematics during game play		X	ROM, motor control, ataxia, precision, speed		X	
Neuro-QOL	X		Quality of Life	X	X	X
Brief Kinesthesia Test (BKT)		X	Sensory proprioception	X	X	X^b^
Touch Test Monofilaments		X	Tactile sensation	X	X	X^b^
9-Hole Peg Test		X	Dexterity	X	X	X^b^
Montreal Cognitive Assessment (MoCA)		X	Cognitive Screen	X		
Satisfaction Questionnaire		X	Satisfaction with treatment received		X	

^a^WMFT is primary outcome measure for pre- and post-testing but is a secondary outcome measure at 6-month follow up.

^b^If participant is able to be physically present for administration.

#### Primary outcome measures

Qualitative analysis of feedback from our Advisory Board indicated two main therapy objectives: 1) regaining sufficient motor control to accomplish daily tasks/hobbies independently (using both hands) and 2) decreasing the time/effort required to perform tasks.

To address the former, quality of arm use for daily activities is measured via the (MAL, which is a reliable and valid measure of quality of movement and arm use in individuals with subacute stroke [45]). To address stakeholders’ priority of increased speed of movement, the WMFT measures the time to complete standardized functional movements [42, 46–48]. It has established reliability and validity [42, 46–48] and has been commonly employed in previous CI therapy trials [5, 19, 37], providing a metric against which to compare the results of the proposed trial. The WMFT is listed as a primary outcome measure during the treatment phase, but as an exploratory outcome in follow-up due to limitations in capturing this measure remotely should attrition be problematic (e.g. participants moving away, losing transportation, etc.).

#### Exploratory outcome measures

Secondary/exploratory outcome measures are measures which a) have yet to be validated (e.g., kinematic data obtained from new wearable sensors) or whose psychometric properties are still being established (e.g., Neuro-QOL), b) measure constructs that may impact response to the therapy (e.g., sensation, cognition), c) may be less sensitive to treatment change (e.g. 9-Hole Peg Test) [49], or d) measure potential moderating factors such as compliance.

The Quality of Life in Neurological Disorders (NeuroQOL) assessment is a validated computerized adaptive self-report measure of health-related quality of life for individuals with neurological disorders. It assesses aspects of physical, cognitive, emotional, and social functioning that are important to stakeholders and was developed using patient-centered methods [50–52].

Wearable sensors quantify both arm use and kinematics of arm movement throughout participants’ daily activities. These sensors are worn bilaterally on the wrists and upper arms, as well as on the chest. They quantify arm position using integrated accelerometer, gyroscope, and magnetometer sensors. Participants are instructed to wear their activity monitors on both arms for two days prior to treatment initiation in order to establish a baseline measure of arm use. They wear the activity monitor every day during the intervention phase of treatment. Data from these devices has not yet been clinically validated; the current study plans to examine the utility of this objective data as an exploratory analysis given the strong need for reliable, objective measures of daily arm use. Angles of the shoulder, elbow, and trunk can be derived from sensor outputs. Additionally, kinematic data collected during game play (participants in Groups 2 and 3) will be similarly examined. These data provide x,y,z coordinates of Kinect skeletal joints. Kinematic metrics that can be examined from both sets of data include speed of movement, smoothness of movement, and range of motion. One range of motion metric particularly relevant to stakeholders is Kinematic Reaching Volume, defined as the percentage of space that an individual accesses (convex hull of observed movements) relative to the volume that could be accessed if full range of motion were present.

The Brief Kinesthesia Test (BKT) quantifies error in targeted reaching to evaluate kinesthetic impairment [53]. It can sensitively detect differences in kinesthetic sense between people with mild-moderate hemiparesis post-stroke and age-matched controls [54].

Touch Test™ monofilament aesthesiometer quantifies the threshold of index finger touch perception in grams [55] with acceptable interrater reliability (ICC = .77–.99) [56] and test-retest reliability (ICC = .69–.71) [57]. Touch test data will be log transformed as recommended for normalization [58].

To enable examining individual factors associated with treatment response (secondary objective), the following demographic variables and treatment related factors will be collected: gender, race/ethnicity, premorbid handedness (self-report), rural/urban status according to census data [59], gross cognitive ability via the Montreal Cognitive Assessment [60–62], total hours of mitt use (Group 1), total hours of game play (Groups 2 and 3), number of computerized MAL administrations (Groups 2 and 3), and number of repetitions. Number of repetitions will be measured via therapist logging for Group 1, video review for Group 4, and in-game logging for Groups 2 and 3. In-game movements are defined as identified gestures that yielded an in-game action (e.g., moved the kayak forward).

#### Standardization of assessments

Project testers undergo extensive training on the measures by an experienced tester prior to testing any participants. They complete two video-taped testing sessions to enable their intra-rater reliability to be established. During these testing sessions, an experienced tester will also rate “participant” performance to enable establishing inter-rater agreement. If agreement is less than excellent (e.g., discrepancies of >.3 on individual WMFT performance time item scores), additional training is required. All testing sessions will be video-recorded for later reference and fidelity review.

### Participant timeline

Four centers will randomize 224 individuals. Enrollment began March 2016. The first baseline assessments were conducted during the third week of March 2016. Anticipated duration of data collection will be 2.5 years. Participation will occur over six months for individuals randomized to Groups 1–3. Individuals in Group 4 participate for approximately seven months.

An initial phone screening is often conducted to screen for potential eligibility. Participants deemed potentially eligible for the study based on the phone screening complete an in-person screening at their respective clinical site. Eligible individuals who have provided informed consent will be randomized. Enrollment is defined as having signed the Informed Consent document. Prospective participants who express interest are scheduled to complete screening with their respective site coordinator. Methods of recruitment are described below.

**Fig. 2:**
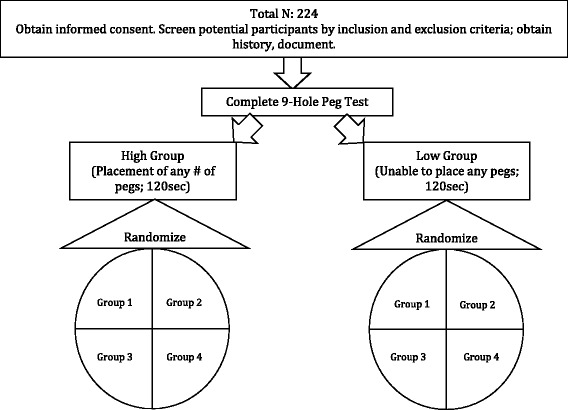
Schematic illustrating the randomization process

Participants deemed eligible to participate following initial screening (see In Person Screening Form on ResearchGate) are stratified into two functional groups (higher and lower functioning) prior to randomization (Fig. 2). Stratification is based on the participants’ ability to place any number of pegs on the 9-Hole Peg Test within 120 s. Individuals are classified as higher-functioning if they can place any number of pegs (1–9) within the allotted time. If unable to place any pegs, the participant is classified as lower-functioning. Stratification serves to more equally distribute the initial motor functioning of participants across groups. This is important because response to motor training depends, in part, on the extent of dexterous function of the participant [63, 64]. The sequence for randomization is represented in Fig. 2. Table 5 outlines the study structure.

**Table 5:** Study Structure

**STUDY ARM**	**Pre-tx testing** Week 1	**THERAPY** (Weeks 2–4)	**Post-tx testing** Week 5	**6 month follow-up testing**	**THERAPY** (Group 4 only; crossover for 3 weeks)
**Group 1** **CI therapy** (*n* = 51)		35 face-to-face hours			
**Group 2** **CI therapy gaming** (*n* = 51)		5 face-to-face hours *in clinic*, 15 h in-home gaming			
**Group 3** **Telerehab** **CI therapy gaming** (*n* = 51)		5 face-to-face hours *in clinic*, 4 face-to-face hours via video conference, 15 h in-home gaming			
**Group 4** **Standard Upper Extremity Rehabilitation** (*n* = 51)		5 face-to-face hours			2 h consultation in clinic, 15 h in-home gaming followed by post-treatment testing

### Sample size

A sample size was selected to provide 80% power to detect a minimal clinically important difference (MCID) in improvement on the WMFT between traditional CI therapy and therapist-as-consultant in-home gaming CI therapy. According to Lin & co-authors [65], the MCID on the WMFT is a 16% change. Taub and colleagues found that subjects undergoing traditional CI therapy exhibited an average improvement of 20.5% from pre-test to post-test on the WMFT [13]. A power analysis, using a Monte Carlo approach, indicated that 51 subjects would be sufficient. The analysis was based on the data from a previous study [13] and used a two-sided test and an alpha level of 0.05. The CI therapy literature shows that the effect size for changes in real-world arm use is approximately three times larger than the effect size for changes in WMFT score [13]. This suggests that a study that is adequately powered to detect minimally clinically significant changes in the WMFT will also be sufficiently powered to detect changes on the MAL. Accounting for an estimated 10% attrition, we anticipate enrolling 224 subjects into the study among the four sites.

### Recruitment

Prospective participants will be identified largely through mining electronic medical records for ICD-9 and ICD-10 codes that indicate potential eligibility (Table 5). Potentially eligible individuals will then be sent a direct mailing via United States Postal Service with an informational brochure and contact information for their local study representative. Recruitment will also target community groups, the general clientele of outpatient clinics, discharge materials on inpatient rehabilitation units, and relevant community organizations. This recruitment approach attempts to reduce selection bias through active pursuit rather than passive acceptance of highly motivated participants already actively seeking participation in research studies.

### Assignment of interventions

*Sequence Generation and Allocation Concealment Mechanism:* Each site will keep two separate large envelopes, one for the low functioning group and one for the high functioning group. There will initially be 3 sets of each group number (1, 2, 3, 4) in each envelope. The group numbers are sealed (e.g., written on folded over sticky notes or within small sealed envelopes) such that participants (as well as research coordinators) are unable to see the numbers when participants are self-selecting a group. Low functioning participants will choose a number from the “low” envelope and higher functioning participants will chose a number from the “high” envelope. The number selected will not be placed back into the envelope. When there are 5 numbers left in an envelope, 2 more of each number will be added by the research coordinator prior the randomization of the next participant. This will ensure complete randomization of all participants, while enhancing probability of evenly balanced group sizes.

#### Implementation

Randomization is overseen by each site’s research coordinator. Participants self-select a sealed group allocation from the envelope.

#### Blinding

Tester(s) are blinded to participant group assignments. Participants, interventionists, and study administrators have access to study group information. Participants are instructed not to disclose any aspects of their treatment to the tester in order to maintain blinding.

### Data collection & management

#### Data collection

All non-electronic data (e.g., signed consent forms, hard copies of assessment forms) are stored in a secure, locked filing cabinet behind a locked door at each study site. Testing data are entered into a secure (and backed up) REDCap [http://projectredcap.org] database within 48 business hours of data collection. Electronic data are collected from the motion capture wearable devices, the gaming system, and NeuroQOL computerized adaptive assessment. All electronic data are stored in an electronic repository behind The Ohio State University Medical Center’s firewall according to participant ID#. Video data are sent from study sites to The Ohio State University (OSU) via encrypted USB storage drives. Identified (coded) study records are retained until analysis is completed on the project. All electronic data is stored in REDCap for a minimum of seven years after study completion. After publication of research findings, de-identified records will be published open-source on ResearchGate.

Pre-, post-, and 6-month follow up testing appointments are scheduled by the research coordinator at the time of intervention scheduling. Data to be collected includes potential adverse events, sensorimotor outcome measures, and client satisfaction questionnaire (see Table 6 below). Data collection forms specific to this study can be found on the PI’s ResearchGate account.

**Table 6:** ICD-9 and ICD-10 codes describing hemiplegia/monoplegia secondary to stroke

Diagnosis	ICD-9 Code	ICD-10 Code
Cerebral Infarction, unspecified	434.91	I63.9
Hemiparesis/hemiplegia	342.90, 438.20–22	G81.90, I69.359, I69.351, I69.354, I69.159
Monoparesis/monoplegia	344.40, 344.5	G83.30–34, G83.20–24, I69.939
Flaccid hemiparesis/hemiplegia	342.00–02	G81.00
Spastic hemiparesis/hemiplegia	342.10–12	G81.10–14
Monoplegia of upper limb	344.40–42	G83.20–24

To enhance participant retention, a financial incentive of $50 per occasion is given for attending testing sessions. A thank-you mailing is sent to participants a month after study completion with reminder of their follow-up testing date. Additionally, members of the Advisory Board suggested the following compliance-enhancing measures to be employed in this trial: 1) frequent feedback regarding adherence, 2) reminder phone-calls (to attend in-person therapy, complete scheduled at-home game play, and perform home exercises), 3) providing an instructional DVD for the client and family (e.g., to educate/inform family members about CI therapy), and 4) being provided with a T-shirt at study completion if >90% adherence was achieved. Inevitable disruptions (e.g., due to illness) of up to 5 treatment days will be permitted and treatment extended accordingly until all treatment sessions have been completed.

#### Data management

Data collection and accurate documentation are the responsibility of study staff under the supervision of the PI. All study documentation is reviewed by the data entry staff, ensuring that they are accurate and complete. Checklists are utilized to minimize missing data resulting from improper staff procedure.

#### Quality control procedures

Participant game-play actions are logged. The game records the x-y-z coordinates of each skeletal joint (skeletal data). It also records gestures and game actions that were detected, each with time-stamps that can be used to determine total play time. MAL and problem-solving responses are also recorded. These data are stored locally until it is periodically pushed to a cloud server. A report is automatically generated from these files, including MAL scores and active play times. This report is accessed by the therapist during consultation visits to trouble-shoot barriers to adherence if needed.

Data entry is performed by Research Assistants (RAs) at OSU. Data entry errors are minimized by training data entry personnel on how to administer the outcome measures. Data entry personnel also review the dataset for outliers and improbable values every 3 months to identify possible data entry errors. If aberrant values are observed, these are cross-referenced with the original forms and video-taped testing sessions as necessary. Neatness of documentation is stressed to the testers to ensure that forms are easily legible to those who are entering data. Paper forms are scanned and stored on the secure backup server for easy access by any member of the OSU project staff.

### Statistical methods

#### Aim 1

*Compare the effectiveness of two technology-based models of in-home CI therapy* versus *traditional clinic-based CI therapy* versus *standard upper extremity rehabilitation for improving upper extremity motor function.* Therapy-induced changes in performance speed (WMFT), real-world arm use (MAL), and quality of life (NeuroQOL) will be analyzed via mixed effects linear models. Initially, separate models will be constructed for the three primary outcome measures. Each of these models will include treatment and time (as well as their interaction) as fixed effects and participant as a random effect. In these models, the interaction of treatment and time is the primary effect of interest, since it tests the difference in change over time among the four treatment groups. The response variables will be transformed to a natural log scale before model fitting to aid in interpretation such that the coefficient associated with the time effect within a treatment can easily be interpreted as a percentage change in the outcome variable. Demographic measures and initial scores on outcome variables will be compared between groups using t-tests, non-parametric analogues such as Mann-Whitney U, or chi-squared tests. Significant differences will be considered in interpretation of the results. Results will be reported according to the CONSORT guidelines.

#### Aim 2

*The secondary objective of this project is to promote personalized medicine by examining individual factors that may differentially influence response to one treatment* versus *another.* The following potential factors will be examined for each of the primary outcome variables: mild/moderate versus moderate motor dysfunction (based on ability to place 1 or more pegs on the 9-hole Peg Test at pre-treatment), number of repetitions performed, therapy adherence, tactile sense (Touch Test Monofilaments), proprioceptive ability at baseline (Brief Kinesthesia Test), Kinematic functional reaching volume, cognition (MoCA), PHQ-9 depression score, age, gender, chronicity, and whether or not the dominant hand was more affected. Based on our pilot data (Borstad A, Crawfis R, Phillips K, Pax Lowes L, Worthen-Chaudhari L, Maung D, et al.: In-home delivery of constraint induced movement therapy via virtual reality gaming is safe and feasible: a pilot study, submitted) and data from Taub and colleagues [13], we hypothesize that treatment-induced improvements on our primary outcome variables will be unrelated to the majority of these variables, with the exception of initial motor ability (preserved pinch grasp) [53]. Higher initial motor ability has been associated with 25% and 200% greater improvements in arm use (UAB Traditional CI therapy/ OSU Gaming CI therapy cohorts, respectively), and 25% lesser improvement on the WMFT (both cohorts) [63]. A confirmatory analysis of this hypothesis will be conducted by adding baseline motor ability as a fixed effect to the original mixed effects general linear model. Other exploratory variables will be added to the model in a stepwise manner. Dummy coded contrasts will be utilized for the categorical variables of initial motor ability. Of interest is the extent to which each factor enhances predictive value of the overall model, the interaction of the factor with time, and the 3-way interaction between factor, time, and group. If 3-way interactions are observed, post-hoc tests will be carried out to determine the differential influence of the factor on one treatment versus another, controlling for family-wise error utilizing the Holm method.

#### Missing data

Intent-to-treat analysis will be carried out. In these analyses, data from participants who voluntarily withdraw from treatment, those with poor compliance, and those unable to tolerate the treatment protocol (e.g., increased pain in trained upper extremity) will be included. Pre-treatment scores will be carried over for participants who voluntarily withdraw from the study (conservatively simulating no change pre-treatment to post-treatment). Data from participants who must withdraw for reasons unrelated to the study (e.g., medical issues unrelated to the study) will be removed from analysis via list-wise deletion and will not be included in the intent-to-treat analysis. The mixed effects model planned for analysis is purely likelihood-based and can make use of all of the data available with no necessary modifications for differing numbers of subjects at each time point.

Data from participants lost to follow-up (those who did not complete 6 month assessment) will be imputed via multivariate analysis of prognostic factors. Follow-up data loss is minimized by having primary outcome measures that can be remotely administered when necessary (MAL, NeuroQoL). Missing data will be dummy coded within the general linear model to determine whether data is likely missing completely at random, missing at random, or missing not at random. Alternative methods for addressing missing data will be examined (e.g. maximum likelihood, multiple imputation). Statistical testing will be used to determine whether individuals lost to follow-up differ from those who completed the study.

### Monitoring

#### Data monitoring

The OSU Center for Clinical and Translational Sciences (CCTS) Research Informatics Services Core is used as a central location for data processing and management of electronic data. Electronic data are stored in REDCap for a minimum of seven years after study completion. The provisioning of accounts and user access to specific database(s) is integrated with the OSU Medical Center authentication service for studies containing protected health information (PHI), and the provisioning of access and specific user rights for all studies are managed by CCTS staff. Data safety monitoring is being provided by individuals with appointments at The Ohio State University who have no intellectual/financial stake in the study and who are not otherwise affiliated with the study (independent safety officers). Monthly meetings are planned between project staff and independent safety officers. These individuals also provide determination of severity of adverse events as needed and prompt reporting to IRB.

#### Harms

The Department of Health and Human Services (HHS) Office for Human Research Protections (OHRP) defines unanticipated problems (UP) and adverse events (AE) [66], these definitions are utilized for protection of subjects in the current study. The PI reviews information relevant to the safety of the subjects and the integrity of the data on a monthly basis.

#### Reporting procedures

Therapists administering the interventions assess for adverse events at each clinic visit and report to the site PI, study PI, and the independent safety officer immediately if any adverse event(s) should occur. All reported adverse events are recorded on the Adverse Event Reporting Form (see form on ResearchGate).

To satisfy the requirement for prompt reporting, unanticipated problems are reported using the following timeline:

- Unanticipated problems that are serious adverse events are reported to the IRB within 48 h of the investigator becoming aware of the event.
- Any other unanticipated problem is reported to the IRB within 2 weeks of the investigator becoming aware of the problem if the problem is study-related.
- All other events are reported to the IRB during annual review.

Complaints made by research participants indicating unanticipated risks, participant complaints that cannot be resolved by the research staff, and unapproved changes made to the research to eliminate an apparent immediate hazard to a research participant are also promptly reported. Events related to the research that do not meet the prompt reporting requirements are reported at the time of the continuing review.

#### Auditing

Study staff will permit authorized representatives of PCORI and regulatory agencies to examine (and when required by applicable law, to copy) research records for the purposes of quality assurance (QA) reviews, audits, and evaluation of the study safety, progress and data validity. Additionally, Institutional Review Boards at each study site may at any time initiate an audit.

### Dissemination

The proposed dissemination plan will be further refined during quarterly meetings with the Advisory Board. Board members will participate in generating additional ideas for methods of dissemination. Initial ideas established collaboratively between the Board and academic team include the following: presenting at local support groups, harnessing social media, generating the interest of TV and radio media outlets, and drafting a lay publication to be submitted to StrokeSmart, the publication of the National Stroke Association. To ensure that results are communicated most effectively to the community, members of the Stroke Advisory Board and Co-I Strahl will direct the dissemination activities and will participate in drafting the publication. Select members of the Board have also expressed their enthusiasm with working with the media to share their experiences (and some have already done so).

To increase therapist ownership of this new treatment and to enhance dissemination, we have incorporated community therapists as Advisory Panel Members/Co Investigators on the project. These individuals will provide a valuable perspective regarding how to present these results most effectively to the therapist community. Therapists at the Providence site will also gain practical experience in utilizing this intervention within a rural clinic setting. This site is representative of clinics that serve those (rural) clients who may benefit most from the model of therapy employed here. Therapist stakeholders have indicated their willingness to make the therapist community aware of this new intervention at Continuing Education conferences and through routine clinical interaction.

## Discussion

Motor interventions, such as CI therapy, that include both intensity of training and techniques to enhance carry-over have been shown to enhance post-stroke outcomes and promote brain plasticity [7, 13, 14]. Yet, less intense and less effective approaches are most frequently utilized in clinical care [4]. Moreover, multiple access barriers to quality care exist [32, 67]. The results of this RCT will provide a significant addition to our body of knowledge regarding the potential for functional motor improvement of upper extremity hemiparesis following stroke. Specifically, they will determine whether using technology can address primary access barriers to quality care, including difficulty of travel for in-person therapy, expense, scheduling demands, and dearth of trained CI therapy providers. Moreover, if effective, a remotely-delivered model of care may facilitate patient choice of providers or clinics (e.g. a client may seek a treatment provider who is located far from their home).

## Abbreviations

ADL: Activities of daily living
AE: Adverse event
ARAT: Action research arm test
BKT: Brief Kinesthesia Test
CCTS: Center for Clinical and Translational Science
CI therapy: Constraint-Induced Movement Therapy
CONSORT: Consolidated Standards of Reporting Trials
CVA: Cerebrovascular accident
GCP: Good clinical practice
HHS: Department of Health and Human Services
HIPAA: Health Insurance Portability and Accountability Act
ICD: International Classification of Disease
ICF: Informed consent form
IRB: Institutional Review Board
MAL: Motor Activity Log
MoCA: Montreal Cognitive Assessment
N: Number (typically refers to participants)
NeuroQOL: Quality of Life in Neurological Disorders Scale
OHRP: Office for Human Research Protections
OSU: The Ohio State University
OT: Occupational therapy
PCORI: Patient Centered Outcomes Research Institute
PHI: Protected health information
PHQ-9: Patient Health Questionnaire-9
PI: Principal investigator
PT: Physical therapy
QA: Quality assurance
QC: Quality control
RA: Research Assistant
RCT: Randomized controlled trial
ROM: Range of motion
UAB: University of Alabama at Birmingham
UP: Unanticipated problem
WMFT: Wolf Motor Function Test
